# Case Report: A Fatal Case of Latent Melioidosis Activated by COVID-19

**DOI:** 10.4269/ajtmh.21-0689

**Published:** 2022-02-03

**Authors:** Uday Gulati, Ananya C. Nanduri, Prateek Juneja, David Kaufman, Mindy G. Elrod, Cari B. Kolton, Jay E. Gee, Kristen Garafalo, David D. Blaney

**Affiliations:** ^1^ Department of Internal Medicine, Inspira Medical Center, Vineland, New Jersey;; ^2^ Department of Critical Care Medicine, Inspira Medical Center, Vineland, New Jersey;; ^3^ Department of Infectious Diseases, Cooper University Health, Camden, New Jersey;; ^4^ Centers for Disease Control and Prevention, Atlanta, Georgia;; ^5^ New Jersey Department of Health, Trenton, New Jersey

## Abstract

Melioidosis, endemic in Southeast Asia and Northern Australia, is an uncommon but frequently fatal opportunistic infection caused by the Gram-negative saprophyte *Burkholderia pseudomallei*. We describe the first reported case of activation of latent melioidosis concurrent with COVID-19-associated lymphopenia and neutropenia in the setting of poorly controlled diabetes. A 43-year-old HIV-positive, diabetic man presented to the emergency department with persistent chills and progressive dyspnea. He was admitted for hypoxia. Chest X-ray showed bilateral parenchymal infiltrates suspicious for COVID-19. Shortly after admission, he became acutely encephalopathic, had a generalized seizure, and was transferred to the intensive care unit after intubation. Further workup showed severe neutropenia and lymphopenia. The patient received empiric antimicrobial coverage and was found to be severe acute respiratory syndrome coronavirus 2 positive. He deteriorated rapidly with refractory shock and persistent hypoxemia, and died 40 hours after admission. Blood cultures and sputum cultures obtained via bronchoalveolar lavage returned positive for *Burkholderia pseudomallei*. Given confirmed compliance with antiretrovirals, stable CD4 counts, and no recent foreign travel, the patient likely contracted the *B. pseudomallei* infection from travel to Southeast Asia many years prior and only became symptomatic after succumbing to severe acute respiratory syndrome coronavirus 2 infection. This case highlights the importance of considering activation of latent opportunistic infections by COVID-19 in immunocompromised patients.

## INTRODUCTION

Melioidosis is caused by *Burkholderia pseudomallei*, a Gram-negative, aerobic, motile, non-spore-forming bacillus. *Burkholderia pseudomallei* was previously known to be endemic to Southeast Asia and Northern Australia, but now is emerging globally. ^1^ Acquisition of *B. pseudomallei* occurs from environmental exposure to the bacterium through open lesions on the skin, by inhalation, or through ingestion of contaminated water. The organism can cause acute infections or remain latent within the host for months or years. Clinical manifestations vary from subclinical disease to septicemia. ^2^ Patients with certain risk factors, such as diabetes mellitus, alcohol abuse, chronic renal disease, and chronic lung disease, are at greater risk for acute clinical manifestations and critical illness. ^1^^,^ ^2^ Activation of latent melioidosis has been described among troops returning from Vietnam who became symptomatic in the United States 10 to 20 years after their service. ^3^

## CASE REPORT

A 43-year-old man with a past medical history of insulin-dependent diabetes mellitus and HIV infection presented to the emergency department in late June 2020 for persistent fevers and chills for 1 week. He also complained of fatigue, shortness of breath, diarrhea, and sore throat. He endorsed compliance with antiretroviral therapy. The patient was found to be hypoxic and was subsequently admitted for acute hypoxic respiratory failure. He emigrated from Vietnam to New Jersey in 2014 and was used as a nail technician until the store closed in March 2020 because of the COVID-19 pandemic. His only foreign travel in between was a brief trip to Vietnam in February 2019. When the store reopened in early June 2020, the patient resumed work until mid-June when he started to complain of the presenting symptoms. While quarantining at home per state mandate for persons with flulike symptoms, he began to decompensate, and his family sent him to the emergency department for this evaluation.

**Figure 1.**
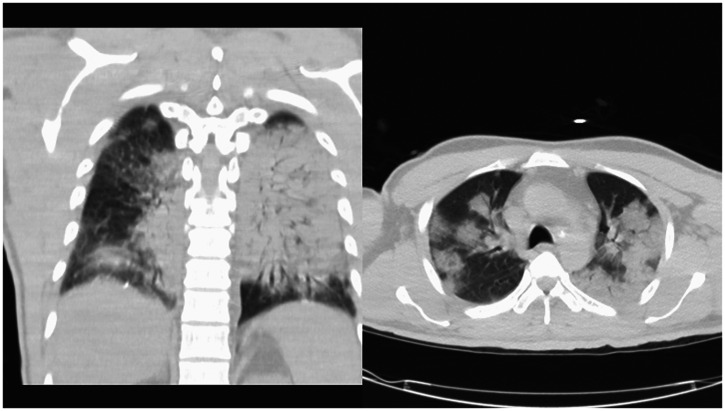
Two views of a computed tomographic scan of the chest without contrast obtained on admission showed extensive bilateral multilobar pneumonia. The diffuse macronodular consolidations and air bronchograms throughout are consistent with bacterial pneumonia.

Shortly after admission, he became acutely encephalopathic, suffered a generalized seizure, and was intubated and transferred to the intensive care workup. His workup was significant for a chest X-ray that showed bilateral parenchymal infiltrates suspicious for COVID-19. Serial chest radiographs within the 3 years prior reported no abnormal findings. A nasopharyngeal swab was collected and sent for severe acute respiratory syndrome coronavirus 2 (SARS-CoV-2) testing via real-time polymerase chain reaction. Most recent outpatient lab results included a CD4+ count of 512 cells/μL and an HIV viral load of less than 40 copies/mL (March 2020), but a repeat CD4+ count and HIV viral load on admission were 103 cells/μL and less than 20 copies/mL, respectively. Laboratory results also showed acute renal and liver failure, high anion gap metabolic acidosis (pH, 6.75; lactic acid, 18.9 mmol/L), and severe neutropenia and lymphopenia. Compared with previous nadirs within the normal range, his absolute neutrophil count decreased from more than 1.6 × 10^3^ neutrophils/µL to 0.3 × 10^3^ neutrophils/µL, and his absolute lymphocyte count decreased from more than 3.3 × 10^3^ lymphocytes/µL to 0.4 × 10^3^ lymphocytes/µL. His hemoglobin A1C levels were consistently greater than 9% during the 3 years prior to presentation. Computed tomography (CT) without contrast showed bilateral multifocal pneumonia, with macronodular consolidations greater on the left than the right (Figure 1). Prior CT of the abdomen obtained in 2014 for left upper quadrant pain did report hypodense areas throughout the spleen, concerning for splenic infarct, infection, or malignancy, but this was not worked up further. Blood cultures were collected before administration of antibiotics or steroids. Empiric antimicrobials were initiated with vancomycin, cefepime, levofloxacin, and the combination of trimethoprim–sulfamethoxazole and steroids given a concern for *Pneumocystis* pneumonia. Anidulafungin was added for treatment of candidiasis. Bronchoscopy, with bronchoalveolar lavage, performed the day after admission showed exanthem and thick yellow secretions throughout the left main bronchus. A SARS-CoV-2 test was positive the day after admission. Remdesivir was not given because of acute renal and liver injury. The patient’s status deteriorated rapidly, with increasing vasopressor requirements and persistent hypoxemia despite appropriate ventilation management, prone positioning, neuromuscular blockade, and high-dose dexamethasone. After discussion with the family regarding his poor prognosis, the patient was declared Do Not Resuscitate and died 40 hours after admission. Both blood and bronchoalveolar lavage cultures returned postmortem grew *B. pseudomallei*, confirmed by the New Jersey Laboratory Response Network laboratory.

## DISCUSSION

It is suspected that our patient was exposed to *B. pseudomallei* while either residing in Vietnam prior to moving to the United States in 2014 or when he visited his hometown near Ho Chi Minh City in 2019. The bacterium can manifest as multiple asymptomatic lesions within visceral organs, and it is possible that the splenic hypodense lesions found on prior CT in 2014 represented latent infection. ^4^ His brother recalled no history of febrile respiratory illness during those times. Genetic analysis of an isolate, BpNJ2020a, obtained from the patient’s culture was performed at the CDC. Multilocus sequencing typing is based on the analysis of portions of seven housekeeping genes, ^5^ and it yielded sequence type 1543, which is represented by one previous example from Vietnam in the pubmlst.org database. ^6^ A much higher resolution method of analysis is based on the examination of single nucleotide polymorphisms of whole genome sequences, and it was conducted as described previously. ^7^ A comparison of the draft genome sequence of BpNJ2020a to a panel of publicly available *B. pseudomallei* genomes indicated that isolate BpNJ2020a clustered with genomes from isolates from Vietnam. The patient’s brother reported that the patient did not have any previous symptoms consistent with melioidosis, nor did outpatient records suggest illness concerning for an acute manifestation of *B. pseudomallei* infection prior to presentation. However, in addition to the risk of exposure from travel to a region endemic to *B. pseudomallei*, the patient had two risk factors for melioidosis: history of alcohol abuse and diabetes. We suspect that the patient succumbed to severe septicemia and refractory acute respiratory distress syndrome (ARDS) from melioidosis resulting from activation of latent *B. pseudomallei* from an inciting event on his immune system. Given confirmed compliance with antiretrovirals evidenced by previously stable CD4+ counts and published observational evidence that HIV infection is not a risk factor for melioidosis, ^6^ the patient likely had latent melioidosis for more than a year and became symptomatic after becoming infected with SARS-CoV-2.

Studies of the pathophysiology of melioidosis suggest that innate and cell-mediated immunity is critical in the long-term control and prevention of acute decompensation from this infection. ^1^ Specifically, patients who are seropositive for the bacterium but who have no clinical history of melioidosis have greater lymphocyte proliferation and interferon-γ production, which arise from natural killer cells and CD8+ T cells, compared with those with a clinical history of melioidosis. ^7^ Fatal melioidosis has also been reported in neutropenic patients, specifically cancer patients receiving chemotherapy. ^8^ Neutropenia increases susceptibility to bacteremia and septic shock, but it is uncertain whether neutropenia is a risk factor for activation of latent melioidosis or presentation with severe acute melioidosis. ^9^ These studies provide evidence of the role different immune responses play in determining the outcome of a *B. pseudomallei* infection.

Serial outpatient laboratory results within the 3 years before the patient’s presentation showed a stable absolute lymphocyte count, absolute neutrophil count, and CD4+T cell count until his admission, when he had a notable decrease in all three, thereby making him prone to acute decompensation from latent melioidosis activation. The patient’s newly compromised innate and cell-mediated immunity, as evidenced by these changes in the blood counts on the date of his admission, was likely a result of COVID-19, as evidenced by his exposure risk and published findings of the association of lymphopenia and neutropenia with COVID-19. ^6^^,^ ^10^ He may have been exposed to the SARS-CoV-2 virus from contact with customers after stores in South Jersey reopened in June 2020, correlating with the onset of his respiratory and systemic symptoms, characteristic of COVID-19. On the date of his admission, the Atlantic COVID Tracking Project reported 180,265 positive cases in New Jersey, supporting his high risk of exposure during widespread community transmission. ^11^ Early during the COVID-19 pandemic, reviews of laboratory abnormalities in hospitalized patients showed that lymphopenia was present on admission in 83.2% of 1,099 patients and that neutropenia was present in 27% of 734 patients who tested positive for SARS-CoV-2. ^6^^,^ ^10^ The exact mechanism by which SARS-CoV-2 causes lymphopenia and neutropenia remains unidentified. Proposed mechanisms for lymphopenia include infiltration and destruction of lymphocytes by SARS-CoV-2 as well as sequestration of lymphocytes at affected target organs. ^12^^,^ ^13^ As it likely did in our patient, lymphopenia in COVID-19 patients has been shown to increase susceptibility to severe bacterial infections, ARDS, and overall mortality. ^13^^,^ ^14^

The patient’s HIV infection and uncontrolled diabetes would make him immunocompromised and thus prone to activation of latent infections such as melioidosis. However, his outpatient laboratory values before his presentation to us make these other etiologies less likely than SARS-CoV-2 to be the cause of his acute decompensation. His pharmacy records showed he was compliant with filling his antiretroviral prescriptions and, until admission, his CD4+ counts remained greater than 500 cells/µL, and his HIV viral load undetectable. Furthermore, despite published evidence that cell-mediated immunity is important in long-term control and prevention of activation of melioidosis, data suggesting that HIV is a risk factor for melioidosis are limited. ^1^^,^ ^7^ Among people with diabetes, uncontrolled hyperglycemia impairs innate immunity, specifically with neutrophils. Published case–control and population-based studies estimate a relative risk of 5.9 to 13.1 for the acquisition of melioidosis in diabetic patients. ^1^^,^ ^2^ Our patient did present with uncontrolled diabetes despite being on a home regimen of 100 U daily insulin, but his high hemoglobin A1c level was unchanged from the previous 3 years. Although poorly controlled diabetes may have contributed to the severity with which the patient presented, he previously showed no clinical evidence of melioidosis. This patient’s recent SARS-CoV-2 infection, with subsequent decreased innate and cell-mediated immunity, more likely led to activation of latent melioidosis.

This case highlights the importance of considering activation of latent opportunistic infections among immunocompromised patients who develop COVID-19. Medical providers should also consider the possibility of activation of latent melioidosis infection when managing COVID-19 patients from endemic areas. Specifically, patients with immunological compromise resulting from COVID-19, such as lymphopenia and neutropenia, may be prone to severe clinical manifestations of melioidosis, such as ARDS and bacteremia, respectively, as described in this case.
